# Efficacy and safety of using Elexacaftor/Tezacaftor/Ivacaftor in the treatment of children with cystic fibrosis: real-world evidence from Brazil

**DOI:** 10.1016/j.jped.2025.05.007

**Published:** 2025-06-25

**Authors:** Guilherme da Silva Martins, Carolina Rambo, Gabriela Spessatto, Maitê Milagres Saab, Bruno Hernandes David João, Aline Didoni Fajardo, Juliana Gonçalves Primon, Thalita Gonçalves Picciani, Roberta Corrêa da Cunha, Herberto José Chong-Neto, Carlos Roberto Lebarbenchon Massignan, Luiz Vicente Ribeiro Ferreira da Silva-Filho, Carlos Antônio Riedi, Nelson Augusto Rosário Filho, Débora Carla Chong-Silva

**Affiliations:** a Universidade Federal do Paraná (UFPR), Divisão de Pneumologia Pediátrica, Curitiba, PR, Brazil; b Faculdade de Medicina da Universidade de São Paulo (FMUSP), Instituto da Criança e do Adolescente, São Paulo, SP, Brazil

**Keywords:** Children, Cystic fibrosis, Cystic fibrosis transmembrane conductance regulator, Elexacaftor, Ivacaftor, Tezacaftor

## Abstract

**Objective:**

Cystic fibrosis (CF) treatment has evolved significantly with the development of CFTR modulators, particularly elexacaftor/tezacaftor/ivacaftor (ETI). This study aimed to evaluate in a real-life context, the efficacy, safety and tolerability of ETI in children and adolescents with CF at a national reference center in Brazil.

**Methods:**

A cohort of 39 patients (mean age: 11.7 years) who had been using ETI for at least three months were evaluated. Anthropometric data, pulmonary function, sweat chloride concentration, pulmonary exacerbations, antibiotic use, and liver function were assessed over a follow-up period of up to 17 months.

**Results:**

Significant improvements were observed in weight Z-score at three months (*p* = 0.046) and six months (*p* = 0.018), as well as absolute weight gain (*p* < 0.001). Height showed absolute growth, but no significant changes in Z-scores. Sweat chloride concentration decreased by 52.8 mmol/L (*p* < 0.001). Pulmonary exacerbations and antibiotic use significantly declined (*p* < 0.001 for both). Despite limitations in spirometry data collection, FEV1 values showed a median increase of 6 percentage points. Oropharyngeal swab cultures for *Pseudomonas aeruginosa* positivity dropped from 43.6 % to 5.1 %. Safety assessments showed a transient rise in alkaline phosphatase (*p* = 0.011), but no significant hepatotoxicity. The most common adverse events were increased respiratory secretions (25.6 %) and abdominal pain (15.4 %). One temporary treatment suspension and one dose reduction occurred, but no patient required permanent discontinuation.

**Conclusions:**

ETI demonstrated effectiveness in improving weight gain, reducing pulmonary exacerbations, and significantly lowering sweat chloride concentration. The treatment was well-tolerated, with a favorable safety profile. These findings align with existing literature, supporting ETI's role as a transformative therapy in pediatric CF management.

## Introduction

Cystic fibrosis (CF) is a rare genetic disorder caused by a series of variants that affect the function of the CFTR gene, which encodes the CFTR protein. The absence or dysfunction of this protein, which forms an ion channel involved in chloride and bicarbonate transport in epithelial cell membranes, leads to various systemic complications, especially in the sweat glands, upper and lower airways, and pancreas, resulting in excessively viscous secretions.^1^^,^^2^

In Brazil, CF prevalence is estimated to vary according to the region, from 1:15,000 to 1:7,500 live births.^4^ More than 2,100 CF-causing variants have been identified to date, classified into six classes based on their impact on CFTR protein production, processing, expression, and function.^2^, ^3^, ^4^, ^5^, ^6^, ^7^

Early diagnosis and strategies of basic CF care that included pancreatic enzyme replacement (PERT), mucolytics, chest physiotherapy and antibiotics transformed the prognosis of CF, with many patients surviving into their third decade of life.^2^, ^3^, ^4^, ^5^, ^6^ This achievement, however, came with a significant burden of treatment and was not uniform in the world, with worse outcomes described among CF individuals from low and middle-income countries (LMICs), such as Brazil.^7^

In recent years, however, a huge advancement in CF treatment has been driven by the development of CFTR modulators — small molecules designed to tackle the basic protein defect. Among them, ETI is a triple CFTR modulator that includes two correctors (elexacaftor and tezacaftor) and a potentiator (ivacaftor). This combination is notable for increasing the availability and functionality of the CFTR ion channel on epithelial surfaces, offering new perspectives for disease management.^4^^,^^8^^,^^9^

Particularly in Brazil, reports of healthcare system experience with ETI treatment are still limited. The pediatric population is particularly significant, as monitoring disease progression in children is not only challenging but also provides the opportunity to prevent the development of some sequelae that begin in childhood.

This study described changes in parameters reflecting treatment effectiveness and the safety of using ETI in pediatric CF individuals eligible for treatment, comparing pre- and post-treatment data collected from medical records at a national reference center.

## Methods

### Study design

This is a longitudinal, prospective, observational study that included retrospective data collection with pediatric patients with CF from the allergy, immunology, and pediatric pulmonology service at CHC-UFPR, Curitiba, Brazil, who had been using ETI for at least three months. The project was approved by the local ethics committee (CAEE: 84822624.0.0000.0096). During the initial patient enrollment for this study, ETI access for all participants was obtained via judicial means, preceding its incorporation into the Brazilian public healthcare system.

Patients who met the inclusion criteria (age 6 to 18 years, with at least one p.Phe508del variant in the CFTR gene) were approached during routine medical consultations, invited to participate in the study, and asked to sign the informed consent form. An exception was made for one patient (age 15) who did not carry a p.Phe508del variant. This patient, with CFTR variants p.Phe316Leu and p.Arg117Cys, initiated ETI via a court-authorized judicial request based on disease severity and anticipated clinical benefit, despite these variants not being covered by the standard Brazilian indication criteria at the time.

Patients who were not undergoing treatment with ETI due to eligibility criteria related to age or absence of the p.Phe508del variant, as well as those with incomplete exam data, were excluded. The retrospective nature of this study and reliance on historical diagnostic reports precluded the uniform ascertainment of precise c.DNA nomenclature for all CFTR variants. Therefore, variants are presented based on the available documentation, which predominantly utilizes protein-level or legacy nomenclature.

Data were obtained from the patient's medical records. Study outcomes included changes in Z-score levels of weight, sweat chloride concentration, pulmonary function and exacerbations, antibiotic use, oropharyngeal swab cultures, liver function, and adverse effects.

### Weight and height

The weight and height gains were calculated based on the specific reference values for biological sex and age from the World Health Organization's (WHO) Z-scores and were measured immediately before the start of ETI treatment, as well as at three and six months afterward.

### Sweat chloride

The results collected were from the test performed at diagnosis or the most recent test before the initiation of ETI treatment, and again after at least three months of treatment. Sweat testing was performed by using pilocarpine and electric stimulation for sweat collection, and chloride dosage by coulometry using a chloridometer, as recommended.^1^

### Pulmonary function

The JAEGER Vyntus™ PNEUMO flow spirometer was used to perform the lung function tests. In addition to the children's anthropometric data, information such as sex and age was recorded in the system to adjust the reference values. The values ​​developed for the Brazilian population by Pereira et al.,^10^ were used as references for predicted values ​​of forced expiratory volume (FEV1), forced vital capacity (FVC) and forced expiratory flows 25 %−75 % (FEF 25 %−75 %). Brazilian reference values were used for cohort-specific appropriateness, as international standards could underestimate impairment in this population. The spirometry values closest to the start of ETI treatment were considered, and the measurements were repeated after at least three months of treatment.

### Pulmonary exacerbation (PEx)

The number of pulmonary exacerbations (PEx) was assessed through routine consultations with the patients, who were also regularly asked about their health status between visits. A PEx was defined as an acute respiratory worsening from the patient’s baseline pattern,^11^ confirmed by the attending physician’s clinical judgment considering reported symptoms, physical examination, and, when relevant, radiological changes, typically requiring a change in management.

The number of exacerbations was collected from the period up to one year before the start of ETI treatment and after ETI use, with the annual pre-ETI baseline chosen to account for potential seasonal variations in exacerbation rates. An annual average (annualization) was calculated for statistical comparison purposes.

### Antibiotic use

The number of antibiotics used was assessed through routine consultations with the patients, who were also regularly asked about antibiotic use between visits. Antibiotic use was defined as any documented course of systemic antibiotics (oral or intravenous) prescribed for an acute respiratory event, irrespective of the need for hospitalization. Data was collected from the period up to one year before the start of ETI treatment and after ETI use. An annual average (annualization) was calculated for statistical comparison purposes.

### Microbiological surveillance

Airway microbiological surveillance is routinely requested in this service during follow-up consultations. It was conducted using oropharyngeal (throat) swabs, a standard and well-accepted collection method for pediatric patients in the present service. Samples from all patients were consistently collected using this method by the same trained team and processed at the same microbiology laboratory. Data regarding the presence (positive or negative) of *Pseudomonas aeruginosa* were collected from patient records for the year prior to ETI initiation and during ETI use. The presence was categorized as either negative or positive during the analyzed period.

### Liver function

Liver function was analyzed through routine laboratory tests conducted for patients in the present service. As recommended for CF patients, safety data was collected, including levels of aspartate aminotransferase (AST), alanine aminotransferase (ALT), gamma-glutamyl transferase (GGT), alkaline phosphatase (ALP), and albumin. These values were gathered from up to one year before the start of ETI treatment and after at least three months of treatment.

### Adverse effects

The presence of adverse events was assessed through routine consultations with the patients, who were also regularly asked about the occurrence of these events between consultations. The events monitored after the use of ETI included increased respiratory secretions, headache, abdominal pain, acne, diarrhea, vomiting, or death, with other events potentially being reported as well.

### Statistics

Data analyses were conducted using Microsoft Excel® and SPSS version 29.0.2. Quantitative variables were described using mean, standard deviation, median, and minimum–maximum values. Categorical variables were summarized as absolute and relative frequencies (percentages), and comparisons were performed using the binomial test where appropriate. For comparisons between two evaluation time points (pre- and post-ETI) for quantitative variables, either the paired Student's *t*-test or the non-parametric Wilcoxon signed-rank test was utilized, with the choice depending on data distribution. The assumption of normality for parametric testing was assessed using the Kolmogorov–Smirnov test. All inferential tests were bilateral, and a *p*-value < 0.05 was considered statistically significant.

## Results

### Epidemiological and genetic profile

This study included 39 patients, with a mean age of 11.7 years, who had been using the triple modulator ETI for at least three months until the data collection was completed. The baseline characteristics of the participants are outlined in Table 1. The majority of the population was male (22, 56.4 %), and only one patient did not carry any p.Phe508del variant (1, 2.51 %). Among the participants, 19 (48.7 %) were homozygous for this variant. The median duration of treatment was 9 months, ranging from 3 to 17 months, with 12 (30.8 %) patients having previously used the double modulator lumacaftor/ivacaftor; no patient in this cohort had prior exposure to ivacaftor monotherapy.

**Table 1:** Patients' epidemiological and genetic profile.

Variable	Classification	*n*	%
Age (years)	Mean ± SDS (minimum-maximum)	11.7 + 2.8 (7–18.5)	
Biological sex	Female	17	43.6 %
	Male	22	56.4 %
CFTR variants			
	F/F (p.Phe508del / p.Phe508del/)	19	48.7 %
Second variants	F/non-F (p.Phe508del / second variant)		
	p.Gly542*	4	10.2 %
	p.Ser4*	3	7.69 %
	p.Arg1162*	3	7.69 %
	p.Tyr1092*	2	5.12 %
	p.Arg117Cys	1	2.51 %
	p.Arg347Pro	1	2.51 %
	p.Ala455Glu	1	2.51 %
	p.Ile506del	1	2.51 %
	p.Ala561Glu	1	2.51 %
	pThr1179llefs*17	1	2.51 %
	Deletion of exons 2 and 3 (ENST00000003084)	1	2.51 %
	non-F / non-F (p.Gly85Glu/p.Gly542*)	1	2.51 %
Duration of use of ETI (months)	Median (minimum–maximum)	9 (3–17)	
Another CFTR modulator before ETI (Lumacaftor/ Ivacaftor)	Yes	12	30.81 %

CFTR, cystic fibrosis transmembrane conductance regulator; ETI, elexacaftor/tezacaftor/ivacaftor; SDS, standard deviation score.

### Effectiveness

Table 2 presents the estimates of treatment effectiveness in terms of Z-Scores for weight and height gains and sweat chloride concentration.

**Table 2:** Variations from baseline in key effectiveness metrics (weight Z-score, weight in kilograms, height Z-score, height in centimeters, and sweat chloride concentration in mmol/L).

Variable	Time compared to ETI use	Mean ± SDS	Median (min-max)	Difference compared to baseline	*p* value^a^
Weight Z-score	Baseline (*n* = 31)	−0.71 ± 1.69	−0.59 (−6.6; 1.99)		
	Month 3	−0.55 ± 1.59	−0.21 (−5.81; 1.92)	0.16	**0.046**
	Baseline (*n* = 27)	−0.78 ± 1.26	−0.59 (−3.53; 1.40)		
	Month 6	−0.52 ± 1.20	−0.21 (−3.04; 1.43)	0.26	**0.018**
Weight (kg)	Baseline (*n* = 31)	33.7 ± 13.5	30 (15.8; 72)		
	Month 3	36.4 ± 13.9	33 (16.9; 73)	2.7	**<0.001**
	Baseline (*n* = 27)	34.7 ± 13,0	33 (17.7; 72)		
	Month 6	38.4 ± 13.3	36 (18.9; 74)	3.7	**<0.001**
Height Z-score	Baseline (*n* = 31)	−0.57 ± 1.24	−0.83 (−3.64; 1.61)		
	Month 3	−0.62 ± 1.38	−0.50 (−4.92; 1.98)	−0.06	0.661
	Baseline (*n* = 27)	−0.56 ± 1.10	−0.77 (−2.87; 1.18)		
	Month 6	−0.47 ± 1.06	−0.48 (−2.34; 1.46)	0.09	0.437
Height (cm)	Baseline (*n* = 31)	138.0 ± 17.0	138 (108.5; 168)		
	Month 3	140.1 ± 16.59	141 (111; 171)	2.1	**<0.001**
	Baseline (*n* = 27)	141.0 ± 15.8	145 (112; 168)		
	Month 6	144.6 ± 15.3	149 (113; 172)	3.6	**<0.001**
SCC (mmol/L)	Baseline (*n* = 19)	93.2 ± 14.4	93 (66; 128)		
	After ETI	40.4 ± 17.5	40 (15; 69)	52.8	**<0.001**

Cm, centimeters; ETI, elexacaftor/tezacaftor/ivacaftor; kg, kilograms; SCC, sweat chloride concentration; SDS, standard deviation score.

*p* values in bold: results with statistical significance.

a Paired Student's T-test, *p* < 0.05.

All 39 patients included in the study had their weights and heights measured during consultations, with Z-Scores calculated. Statistically significant weight gain was observed both in Z-Scores (at three months, *p* = 0.046; and six months, *p* = 0.018) and in absolute weight (kilograms) (at three months, *p* < 0.001; and six months, *p* < 0.001). However, no significant increase in height was observed in Z-Scores within this sample, although absolute height (centimeters) showed significant improvement at the third (*p* < 0.001) and sixth (*p* < 0.001) months.

Of the total sample, 19 patients (48.7 %) were able to undergo a second sweat test, with limitations due to logistical, spatial, or scheduling issues with the specialized center conducting the test. A significant reduction of 52.8 mmol/L in sweat chloride concentration was observed compared to baseline values (*p* < 0.001).

Table 3 further demonstrates treatment effectiveness, focusing on respiratory and airway parameters, including pulmonary function as measured by FEV1 values in spirometry, pulmonary exacerbations, and subsequent antibiotic use. Of the total, 10 patients (25.6 %) were able to perform a reliable spirometry test after the initiation of treatment. One patient (2.6 %) had no recorded data on pulmonary exacerbations or antibiotic use in their medical records.

**Table 3:** Variations from baseline in key effectiveness metrics of respiratory and airway parameters (pulmonary function, pulmonary exacerbation, antibiotic use).

Variable	Time compared to ETI use	Mean ± SDS	Median (min-max)	Difference compared to baseline	*p* value^a^
FEV1 (%)	Baseline (*n* = 10)	76.3 ± 20.7	75.5 (38-114)		
	After ETI	75.8 ± 27.2	81.5 (11-105)	- 0.5	0.192
Pulmonary exacerbations (Pex)	Baseline (*n* = 38)	1.3 ± 1.6^b^	1 (0-6)		
	After ETI	0.4 ± 0.7^b^	0 (0-3)	- 0.9	**<0.001**
Antibiotic use	Baseline (*n* = 38)	1.5 ± 2.1^b^	1 (0-10)		
	After ETI	0.5 ± 1.0^b^	0 (0-4)	- 1.0	**<0.001**

ETI, elexacaftor/tezacaftor/ivacaftor; FEV1, forced expiratory volume in 1 second; kg, kilograms; SDS, standard deviation score.

*p* values in bold: results with statistical significance.

a Wilcoxon signed-rank test, *p* < 0.05.

b Annual average.

Oropharyngeal swab cultures were routinely requested at each follow-up appointment, ensuring that all 39 patients underwent testing. Comparatively, 17 patients (43.6 %) tested positive for *P. aeruginosa* bacteria up to one year before initiating ETI. Following treatment, only 2 patients (5.1 %) had bacterial presence in their cultures at any point thereafter. This study did not differentiate the cultures concerning intermittent infection or chronicity.

### Safety and tolerability

Table 4 presents liver function tests as metrics reflecting treatment safety. A slight, yet statistically significant, increase in the average ALP levels of 67.5 U/L (*p* < 0.0)^11^ was observed.

**Table 4:** Variations from baseline in key safety metrics of liver function (aspartate aminotransferase, alanine aminotransferase, gamma-glutamyl transferase, alkaline phosphatase, and albumin).

Variable	Time compared to ETI use	Mean ± SDS	Median (min-max)	Difference compared to baseline	*p* value^a^
AST	Baseline (*n* = 26)	29.6 ± 8.6	30 (17; 49)		
	After ETI	33.8 ± 14.4	29 (19; 72)	4.2	0.082
ALT	Baseline (*n* = 25)	30.8 ± 18.8	23 (11; 81)		
	After ETI	36.8 ± 33.3	28.5 (13.7; 168)	6.0	0.520
GGT	Baseline (*n* = 23)	23.8 ± 14.1	20 (9; 71)		
	After ETI	25.3 ± 24.6	19 (8.8; 132)	1.5	0.884
ALP	Baseline (*n* = 21)	250.2 ± 88.5	257 (60; 395)		
	After ETI	317.7 ± 120.8	319 (93; 494)	67.5	**0.011**
Albumin	Baseline (*n* = 16)	4.5 ± 0.3	4.5 (4.1; 5.1)		
	After ETI	4.5 ± 0.3	4.5 (4; 4.8)	0	0.970

AST, aspartate aminotransferase; ALT, alanine aminotransferase; GGT, gamma-glutamyl transferase; ALP, alkaline phosphatase; ETI, elexacaftor/tezacaftor/ivacaftor; SDS, standard deviation score.

*p* values in bold: results with statistical significance.

a Wilcoxon signed-rank test, *p* < 0.05, for AST, ALT, GGT, and ALP; Paired Student's *t*-test, *p* < 0.05, for Albumin.

The most common adverse effects reported were increased respiratory secretions (10 children, 25.6 %), abdominal pain (6 children, 15.4 %), headache (3 children, 7.7 %), mild skin reactions (1 child, 2.6 %), diarrhea (1 child, 2.6 %), and vomiting (1 child, 2.6 %). There was one death in the cohort, unrelated to treatment, due to sepsis from an infected wound. One patient temporarily discontinued treatment for one month due to headache, and another had their dose halved for abdominal pain investigation; both subsequently resumed the standard ETI regimen without recurrence of these initial adverse events during the follow-up period.

## Discussion

In the last decade, the treatment of CF has undergone a revolution in terms of pharmacological advancements. Substantial progress has been made with the introduction of therapies that address the primary manifestations of the disease.^2^^,^^10^^,^^12^^,^^14^^,^^15^ The development of CFTR protein modulators has brought unprecedented improvements to the health of CF patients. Among these medications, the triple CFTR protein modulator, ETI, represents a milestone in the history of the disease, combining three small molecules administered orally: elexacaftor, tezacaftor, and ivacaftor.^2^^,^^10^^,^^12^^,^^15^

The present observational study shares the experience of a national reference center in the treatment of children aged 6 to 18 years with CF, providing evidence of the efficacy, safety, and tolerability of CFTR triple modulator treatment, ETI, in the context of its recent approval in Brazil and real-world clinical practice.

The sample described exhibited a relatively uniform distribution regarding age and sex, meeting the criteria for modulator use in Brazil. An exception was made for one patient who did not possess any copies of the p.Phe508del variant but was included following the decision of the healthcare team, who determined that the patient would benefit significantly from the treatment. Existing literature affirms that CF affects individuals of all ethnicities, equally among men and women, a finding consistent with the present sample, which showed a slight predominance of male patients (56.4 %).^10^^,^^14^

The average age of this sample was 11.7 years, ranging from 7 to 18.5 years old. There is a lack of studies on clinical experience with ETI treatment in the pediatric population.^9^ This may be because the combination was only approved by national regulatory agencies in 2022 and incorporated into the public healthcare system in 2023.^16^^,^^17^

Given the data collection period for this study, from January 2023 to July 2024, initially, all patients accessed the medication through judicial means. For data standardization, the collection period was limited to six months of treatment, although some patients had already been using it for up to 17 months by the end of data collection.

The results of this study corroborate previous evidence of ETI's efficacy in improving clinical parameters in patients with cystic fibrosis, including weight gain, reduced exacerbations, and reduced sweat chloride concentration. The benefits observed in the national clinical experience with this treatment were similar to those reported in recent clinical trials or reviews published on pediatric patients.^7^^,^^9^^,^^10^^,^^13^^,^^16^

There was a significant weight gain among patients in the present study. This gain was observed not only in absolute values but also when adjusted for the Z-score of weight, considering age and sex, as recommended by the WHO and the Brazilian Guidelines for the Diagnosis and Treatment of Cystic Fibrosis.^1^^,^^18^

Weight gain is expected and has already been described in previous studies.^4^^,^^9^

It is hypothesized that with the partial or total resolution of CFTR dysfunction, the obstruction of pancreatic ducts by thick secretions and the proper release of digestive enzymes–essential for the digestion and absorption of fats and proteins–is restored.^4^^,^^9^^,^^12^

At this point, the early introduction of ETI seems crucial, as previous clinical studies with adolescents and adults using ETI have shown that reducing enzyme replacement therapy was not possible, justified by the extensive and permanent damage to pancreatic functions in this population.^4^^,^^9^^,^^12^^,^^19^

Another point discussed, which corroborates the weight gain in patients using ETI, is the reduction in caloric needs. This is due to decreased infections and exacerbations, as well as improved respiratory and metabolic expenditure–factors that, when present, prevent weight gain and maintenance.^4^^,^^9^^,^^12^ The patients followed in the present study showed increases in height in absolute values, although they have not yet reached adequate Z-score values. The authors believe that with a longer observation period, this result will be achieved.

As one of the efficacy outcomes, the authors observed a 43.3 % reduction in sweat chloride concentration, with significant values similar to those demonstrated in studies with modulators.^4^^,^^9^ The measurement of sweat chloride concentration, a method developed in 1959, is considered the gold-standard clinical marker for evaluating CFTR function and confirms the diagnosis of cystic fibrosis (CF) in 98 % of cases.^1^^,^^5^^,^^6^ CFTR dysfunction impairs chloride absorption in the ducts of sweat glands, resulting in elevated ion levels in sweat.^1^^,^^5^^,^^6^ CFTR dysfunction represents a spectrum that can vary in severity. Normal chloride levels are below 30 mmol/L; intermediate values range from 30 to 60 mmol/L, and a CF diagnosis is confirmed with chloride values above 60 mmol/L in two samples.^1^^,^^5^^,^^6^^,^^20^^,^^21^

In the present study, the average sweat chloride concentration after a minimum of 3 months of treatment decreased from 93.2 to 40.4 mmol/L, with some patients even reaching normal levels for this test.

Pulmonary function could only be evaluated in 10 cases after the introduction of ETI. Limited access to pulmonary function testing services in patients’ hometowns, as well as technical challenges in performing the test due to age, were factors that contributed to the restriction of this examination.

Cystic fibrosis is a disease that presents a classic obstructive ventilatory pattern on spirometry. The thick secretions perpetuate airway inflammation and, along with chronic bacterial colonization, often lead to irreversible structural changes in the airways of CF patients. The classic functional representation is an obstructive disorder that does not respond to bronchodilators.^22^^,^^23^

The literature describes improvements ranging from 8 to 22 percentage points in FEV1 in patients using ETI.^4^^,^^9^^,^^12^ In the present study, the median variation was 6 percentage points (75.5 % to 81.5 %), a result that should be interpreted with caution given the aforementioned limitations. The modest median FEV1 increase observed, using Brazilian reference values appropriate for this cohort, likely reflects the relatively short follow-up for some patients and baseline FEV1 values that were not severely compromised in many participants.

An important variable evaluated in clinical studies with ETI, especially in the pediatric population, is the reduction in exacerbations and the use of antibiotics.^9^^,^^18^^,^^24^ In the present sample, this reduction was confirmed. There was a general annual average reduction of 69.3 % in pulmonary exacerbations and 66.7 % in antibiotic use, both statistically significant results and similar to those reported in the literature, with reduction rates ranging from 70 to 90 %.^4^^,^^9^ The improvement in ion transport, hydrating secretions and making them less thick and sticky, is the highlight of breaking the inflammation-infection cycle that perpetuates respiratory conditions, often leading to extensive and irreversible pulmonary structural changes.^22^^,^^23^

A 38.5 % reduction in the number of patients with positive oropharyngeal swab cultures for P. aeruginosa was observed after the initiation of ETI (24 cases pre-ETI to 9 cases post-ETI). While this finding is promising, it warrants careful interpretation. The present retrospective analysis documented any positive detection from routine data, without formal classification of chronic versus intermittent colonization, a distinction limited by real-world data collection. Consequently, one or two negative cultures do not necessarily indicate the absence of the bacterial agent or its decolonization.^25^ The literature describes a reduction of approximately 50 % in the detection of these bacteria.^4^

Regarding safety, there is a concern from the manufacturer and based on experience with other modulators, about the potential induction of liver injury caused by ETI, making rigorous liver function monitoring a recommendation in major global protocols.^16^^,^^18^^,^^26^ This monitoring was performed in the patients, and during the follow-up period, no increases in transaminase, bilirubin, or international normalized ratio (INR) levels were observed. An increase in the mean values of alkaline phosphatase was noted after the introduction of ETI, but it occurred in isolation, was transient, showed no clinical correlation, and did not lead to any changes in the therapeutic regimen.

Twenty-two episodes of adverse events were reported, and the temporary increases in respiratory secretions were the most frequent, occurring in 25.6 % of patients, as a reflection of the restoration of CFTR function. Other events considered idiosyncratic, such as headache and abdominal pain, were reported less frequently and without severity. In one case, vomiting led to the temporary suspension of ETI for 15 days. No cases resulted in a longer suspension or discontinuation. Published data indicate mild to moderate adverse effects, rarely requiring suspension or discontinuation.^4^^,^^9^^,^^19^^,^^20^^,^^26^ A real-life study in children using ETI identified headaches as a frequent but non-severe event.^9^

Special attention has been given to events related to mental health in patients using ETI, and significant guidelines have suggested monitoring for depression and anxiety, using validated questionnaires.^17^^,^^27^, ^28^, ^29^

It is already known that patients with CF, even before the era of modulators, faced psychological and social challenges associated with the natural progression of the disease, with reports of distress due to uncertainties, fear, and hesitation caused by frequent clinical fluctuations and the possibility of imminent death.^27^, ^28^, ^29^ Despite this concern, some groups believe that the introduction of the triple modulator ETI and the consequent improvement in quality and life expectancy may reduce anxiety and depression rather than exacerbate them.^26^^,^^29^

The present study presents some limitations, particularly regarding the sample size, which, although representative of studies involving CFTR modulators, may limit the interpretation of certain outcomes. Additionally, the limited availability of reliable follow-up spirometry data for a portion of the cohort, reflecting real-world challenges in pediatric populations (including young age, technical difficulties in maneuver execution pre-ETI, and logistical issues with test access or quality from referring centers), hinders a more comprehensive evaluation of the response in this parameter and underscores the need for improved access to quality spirometry. Furthermore, the assessment of pancreatic function changes post-ETI was beyond the scope of this initial real-world analysis.

Nevertheless, the robust analysis and the use of multiple clinical outcomes in a real-world scenario strengthen the study's conclusions. The inclusion criterion of at least three months of ETI use, while theoretically posing a risk of selection bias by excluding early discontinuations, did not result in such bias in the cohort, as all initiating patients continued treatment beyond this period, reflecting high early adherence.

In conclusion, the present findings suggest that CFTR modulating therapy has proven to be effective and well tolerated, with a favorable safety profile, in pediatric CF patients aged 6 to 18 years who met inclusion criteria. The data underscore the positive impact of ETI therapy in improving clinical outcomes in a real-world setting. These results support the continued use of ETI in the treatment of CF for patients with responsive genotypes, demonstrating significant benefits in this real-world pediatric cohort.

## Conflicts of interest

Débora Carla Chong-Silva, Carlos Antônio Riedi, and Luiz Vicente Ribeiro Ferreira da Silva Filho are occasionally invited by Vertex Pharmaceuticals to give lectures or presentations. However, they are neither employees nor have any employment or ownership affiliation with the company.
